# The Structure of the R2TP Complex Defines a Platform for Recruiting Diverse Client Proteins to the HSP90 Molecular Chaperone System

**DOI:** 10.1016/j.str.2017.05.016

**Published:** 2017-07-05

**Authors:** Angel Rivera-Calzada, Mohinder Pal, Hugo Muñoz-Hernández, Juan R. Luque-Ortega, David Gil-Carton, Gianluca Degliesposti, J. Mark Skehel, Chrisostomos Prodromou, Laurence H. Pearl, Oscar Llorca

**Affiliations:** 1 Centro de Investigaciones Biológicas (CIB), Spanish National Research Council (CSIC), Ramiro de Maeztu 9, 28040 Madrid, Spain; 2 Genome Damage and Stability Centre, School of Life Sciences, University of Sussex, Falmer, Brighton BN1 9RQ, UK; 3 Structural Biology Unit, CIC bioGUNE, Bizkaia Technology Park, 48160 Derio, Spain; 4 MRC Laboratory of Molecular Biology, Francis Crick Avenue, Cambridge CB2 0QH, UK; 5 Structural Biology Programme, Spanish National Cancer Research Centre (CNIO), Melchor Fernández Almagro 3, 28029 Madrid, Spain

**Keywords:** Hsp90 co-chaperone, Pih1, R2TP complex, Tah1, Rvb1, Rvb2, Tel2-Tti1-Tti2, cryo-electron microscopy (cryo-EM)

## Abstract

The R2TP complex, comprising the Rvb1p-Rvb2p AAA-ATPases, Tah1p, and Pih1p in yeast, is a specialized Hsp90 co-chaperone required for the assembly and maturation of multi-subunit complexes. These include the small nucleolar ribonucleoproteins, RNA polymerase II, and complexes containing phosphatidylinositol-3-kinase-like kinases. The structure and stoichiometry of yeast R2TP and how it couples to Hsp90 are currently unknown. Here, we determine the 3D organization of yeast R2TP using sedimentation velocity analysis and cryo-electron microscopy. The 359-kDa complex comprises one Rvb1p/Rvb2p hetero-hexamer with domains II (DIIs) forming an open basket that accommodates a single copy of Tah1p-Pih1p. Tah1p-Pih1p binding to multiple DII domains regulates Rvb1p/Rvb2p ATPase activity. Using domain dissection and cross-linking mass spectrometry, we identified a unique region of Pih1p that is essential for interaction with Rvb1p/Rvb2p. These data provide a structural basis for understanding how R2TP couples an Hsp90 dimer to a diverse set of client proteins and complexes.

## Highlights

• Rvb1p-Rvb2p forms a hetero-hexamer with DII domains recruiting a single Tah1p-Pih1p
• Residues 230–250 in Pih1p are essential to bind Rvb1p-Rvb2p
• 3D structure of yeast R2TP couples an Hsp90 dimer to client proteins
• Tah1p-Pih1p binding to flexible DII domains stimulates Rvb1p-Rvb2p ATPase activity

## Graphical Abstract

**Figure:**
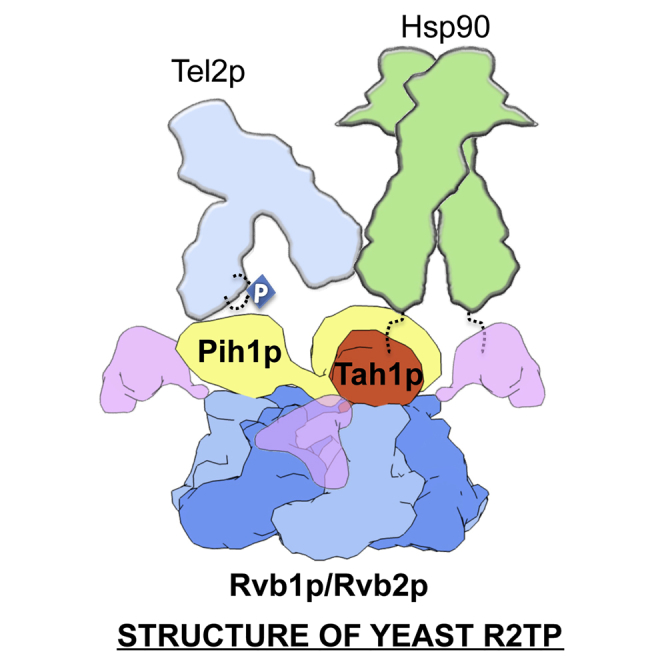

## Teaser

Rivera-Calzada, Pal et al. reveal the 3D architecture of yeast R2TP using sedimentation velocity analysis, cryo-EM, XL/MS, and domain mapping, and find that the Rvb1p/Rvb2p ring binds a single Tah1p-Pih1p unit that modulates Rvb1/2-ATPase activity and couples phosphorylated clients and client adaptors to a single Hsp90 dimer.

## Introduction

Hsp90 is a molecular chaperone implicated in folding and assembly of a large and eclectic set of client proteins and complexes. Specificity is provided by co-chaperones, one of whose functions is to deliver client proteins to Hsp90. Some co-chaperones can themselves be large multi-subunit complexes, adding further levels of complexity in the regulation of Hsp90-mediated folding and assembly (Vaughan, 2014).

R2TP (Rvb1p-Rvb2p-Tah1p-Pih1p in yeast and RUVBL1-RUVBL2-RPAP3-PIH1D1 in humans) is a co-chaperone complex that has been implicated in the stabilization and assembly of phosphatidylinositol-3-kinase-like kinases (PIKKs) (Horejsi et al., 2010, Horejsi et al., 2014, Kim et al., 2013, Pal et al., 2014, Takai et al., 2010), RNA polymerase II (Boulon et al., 2010), and small nucleolar ribonucleoproteins (snoRNPs) (Machado-Pinilla et al., 2012). The AAA+ ATPases Rvb1p and Rvb2p of yeast R2TP assemble as homo- or hetero-hexameric rings, as well as hetero-dodecameric complexes formed by association of two hetero-hexamers. However, in the R2TP complex, it is unclear what is their oligomeric state and how many copies of Tah1p-Pih1p molecules are associated with them (Matias et al., 2015, Nano and Houry, 2013, Vaughan, 2014, von Morgen et al., 2015). This is critically important, as it would influence the number of Hsp90 and client molecules that potentially could associate with R2TP.

Pih1p acts as a central scaffold in R2TP connecting Rvb1p-Rvb2p to Tah1p, which in turn couples the R2TP complex to Hsp90. Pih1p consists of an N-terminal PIH domain and a C-terminal CS domain (Horejsi et al., 2014, Pal et al., 2014). The N-terminal PIH domain binds proteins containing a CK2-phosphorylated motif found, for example, in Tel2p (and human TEL2), a component of the Tel2p-Tti1p-Tti2p (TTT) complex that interacts with PIKK kinases such as Tor, coupling PIKK kinases to R2TP and hence to the Hsp90 chaperone system (Kim et al., 2013, Takai et al., 2010). The CS domain of Pih1p binds the extended C-terminal tail of Tah1p, a small tetratricopeptide (TPR) domain protein that itself binds to the conserved MEEVD C-terminal tail of Hsp90 (Back et al., 2013, Morgan et al., 2015, Pal et al., 2014). The region of Pih1p connecting the PIH and CS domains contains no recognizable structural features but is required for coupling Pih1p to Rvb1p-Rvb2p (Paci et al., 2012).

The AAA+ core of the Rvb1p-Rvb2p hetero-hexamer is formed by domains 1 (DI) and 3 (DIII) from each subunit with Rvb1p and Rvb2p alternating in position around the ring, following 3-fold rotational symmetry (Ewens et al., 2016, Gorynia et al., 2011, Lakomek et al., 2015). Domain II (DII) protrudes outward from the ring and comprises two defined regions, an oligonucleotide-biding (OB) domain (DII exterior) and an internal region closer to the AAA+ ring (DII interior). DIIs are implicated in protein-protein interactions, as seen in yeast SWR1 chromatin remodeling complex (Nguyen et al., 2013) and in Rvb1p-Rvb2p dodecamers (Ewens et al., 2016, Lakomek et al., 2015, Lopez-Perrote et al., 2012). Curiously, cryo-electron microscopy (cryo-EM) and crystal structures of dodecameric Rvb1p-Rvb2p complexes from yeast suggest that all DII domains show a similar conformation when part of the inter-ring interaction (Ewens et al., 2016), and is also observed with human RuvBL1-RuvBL2 (Gorynia et al., 2011, Lopez-Perrote et al., 2012, Matias et al., 2006). In contrast, the structure of Rvb1-Rvb2 dodecamers from the fungus *Chaetomium thermophilum* revealed that the DII domain of Rvb2p substantially rotated with respect to that of Rvb1p (Lakomek et al., 2015). Comparison of these cryo-EM and crystal structures does not clarify the rules directing the conformation of the DII domains and the functional significance of these changes.

Despite substantial structural analysis of its components, the 3D structure and stoichiometry of the assembled yeast R2TP complex is not known. Here, we determine the structure of yeast Rvb1p-Rvb2p at 6.5-Å and yeast R2TP at 8.37-Å resolution, by cryo-EM and single-particle reconstruction. The structures reveal the stoichiometry of the R2TP complex and the role of DII domains in binding Tah1p-Pih1p. Conformational differences between the Rvb1p-Rvb2p hetero-hexamer in isolation and as part of the R2TP or Rvb1p-Rvb2p dodecameric complex, and the effect of Tah1p-Pih1p on Rvb1p-Rvb2p ATPase activity, suggest that the conformation of the DII domain plays an important role in regulating Rvb1p-Rvb2p function.

## Results

### Reconstitution of Yeast R2TP Complex

**Figure 1:**
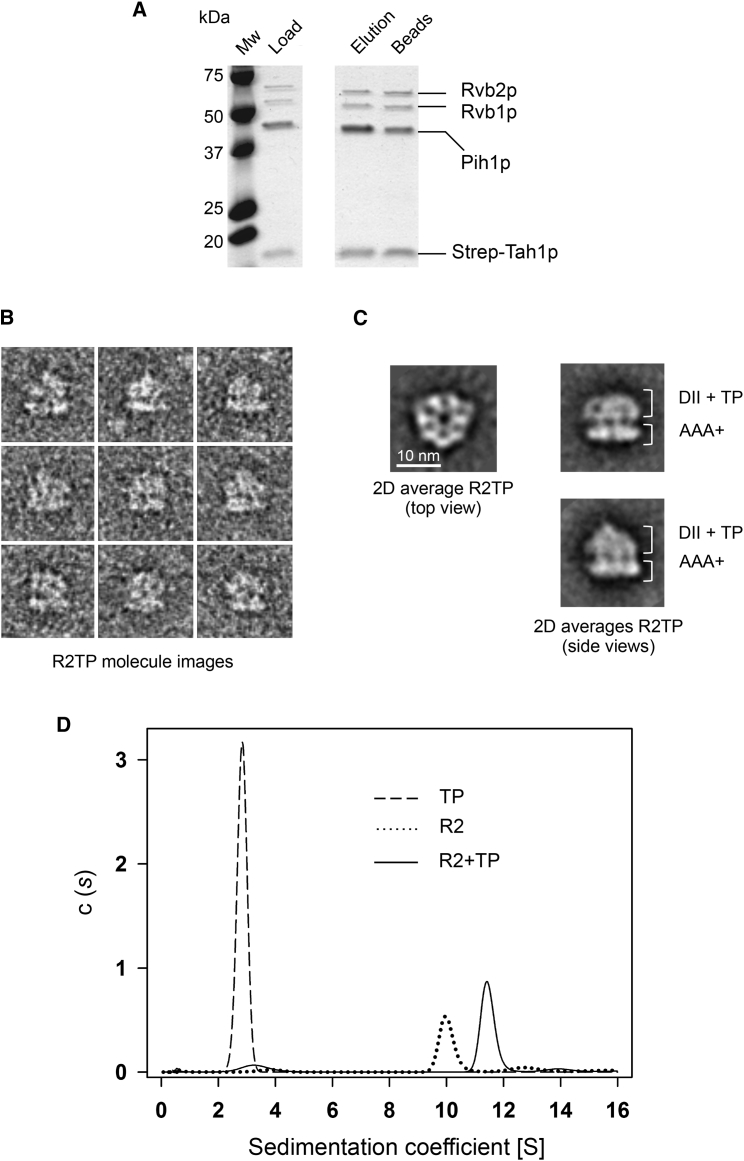
Assembly and Stoichiometry of R2TP (A) Purification of yeast R2TP in pull-down experiments using a Strep-tag in Tah1p. Fractions from the experiment were analyzed using SDS-PAGE. Fractions where R2TP eluted were selected for EM, in some cases after stabilization using glutaraldehyde. Elution, represents material eluted from the pull-down and used in the negative-stain EM experiments. Beads, represents material that remained bound to the beads after the elution. (B) A gallery of single-molecule images of the R2TP complex, after cross-linking, orientated with the projection of the putative AAA+ ring at the bottom. (C) Representative 2D averages of top and side views of the R2TP complex, after cross-linking, obtained by negative staining. Side view averages show a putative AAA+ ring at the bottom, decorated by extra density on top. (D) Oligomerization state determined by sedimentation velocity in an analytical ultracentrifuge. Sedimentation coefficient distribution c(s) profiles corresponding to purified Tah1p-Pih1p (TP) (dashed line), Rvb1p-Rvb2p (R2) complex (dotted line), and the R2TP complex assembled by mixing TP and R2 complexes (solid line). See also Figure S1.

Rvb1p-Rvb2p (R2 hereafter) and Tah1p-Pih1p (TP hereafter) were purified separately as homogeneous sub-complexes. R2TP was reconstituted by mixing R2 with a 10-fold molar excess of TP to saturate all R2 in the mixture and purified by affinity purification via a Strep-tag appended to Tah1p (Figure 1A). The eluted complex was stabilized by mild glutaraldehyde cross-linking and analyzed by EM using negative staining (Figures 1B and 1C). Particles were a mixture of hexameric views perpendicular to the R2 ring and side views where additional density was bound to R2 (Figures 1B and 1C). Side views indicated that R2TP was made of one ring of R2, corresponding to the projection of the AAA+ ring, with extra asymmetric density on one face, henceforth referred to as the top end of the complex.

### Stoichiometry of Yeast R2TP

To facilitate structural analysis of the complex, we determined the oligomerization state of R2 and TP sub-complexes by sedimentation velocity analysis (SV) in an analytical ultracentrifuge (AUC). TP behaved as a single species with an experimental sedimentation coefficient of 2.8S (Figures 1D and S1). Corrected to standard conditions (*s*_20,*w*_ *=*3.0S), this was compatible with a slightly elongated heterodimer (f/f_0_ = 1.6) of 53 kDa molecular mass. For R2, more than 70% of the absorbance in the SV assay corresponded to a 10.0S (*s*_20,*w*_ *=*10.6S) peak consistent with a slightly elongated (f/f_0_ = 1.5) 296 kDa particle, very close to the 306 kDa predicted for a 3 × Rvb1p:3 × Rvb2p complex.

When R2 and TP were mixed (1:2.4 molar ratio), we observed a main peak (>80% of the sample) of 11.5S (*s*_20,*w*_ *=*12.1S) (Figure 1D), corresponding to a molecular mass of 359 kDa, almost identical to the expected molecular mass for a complex composed of one Rvb1p-Rvb2p hetero-hexamer bound to one Tah1p-Pih1p complex (molecular weight, 358.1 kDa). This S value was consistent with the theoretical value (12.05S) calculated from the 3D reconstructions of R2TP.

### Cryo-EM Structure of Yeast R2TP

**Figure 2:**
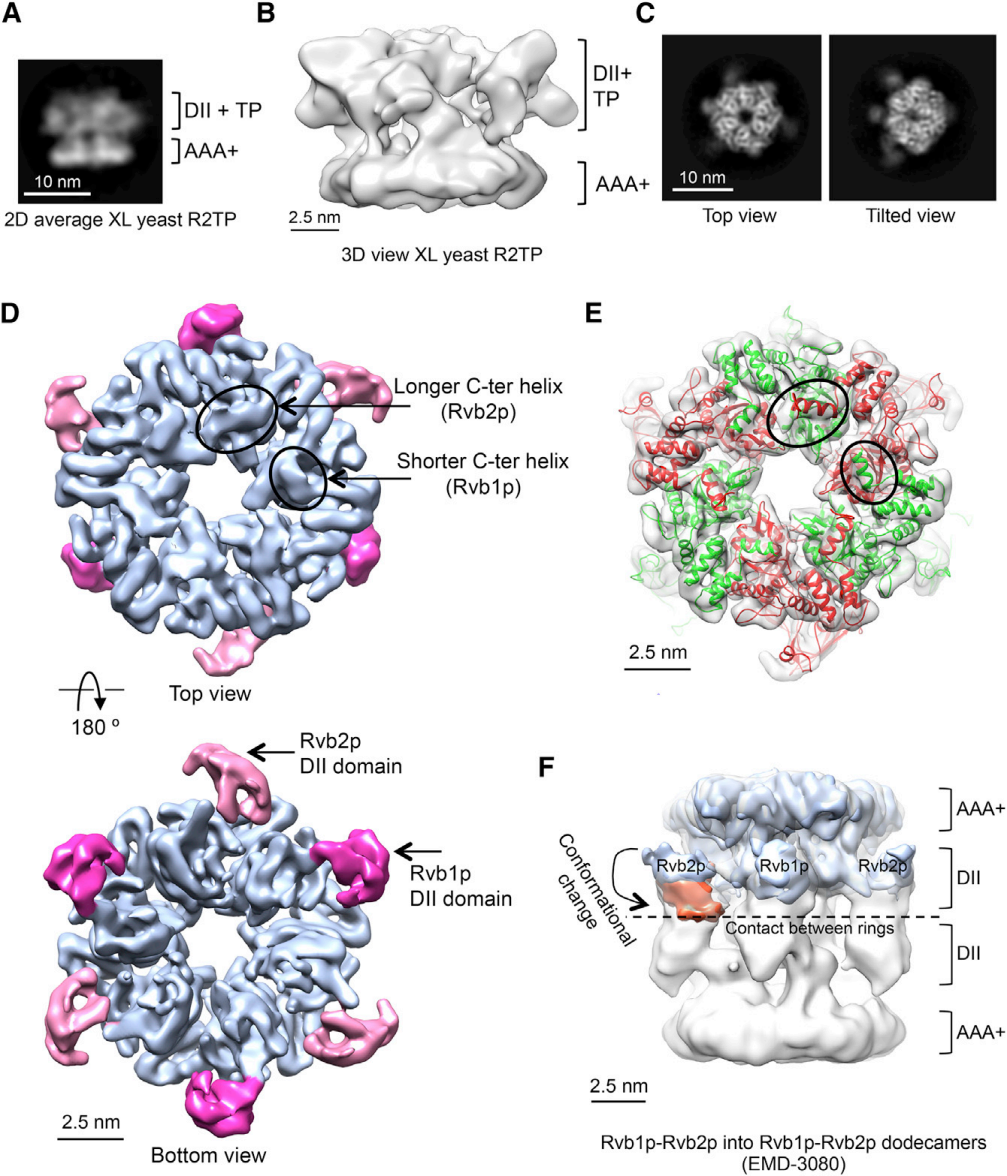
Cryo-EM of Yeast R2TP and Rvb1p-Rvb2p Hexamers (A) Selected 2D average of a side view of stabilized R2TP obtained by cryo-EM. XL stands for cross-linked. (B) One side view of the low-resolution cryo-EM structure of R2TP stabilized using glutaraldehyde. (C) Selected 2D averages of top and tilted views of R2TP (no cross-linking) obtained by cryo-EM. (D) Two views of the 3D structure of R2 hexamers obtained after classification of the total dataset. α helices corresponding to the C-terminal end of Rvb1p and Rvb2p subunits are identified (circled). DII domains in Rvb1p and Rvb2p have been colored in dark and light pink, respectively. (E) Fitting of an atomic model of Rvb1p-Rvb2p within the EM map. Rvb1p is colored in green and Rvb2p in red. α helices at the C-terminal ends of Rvb1p and Rvb2p subunits are indicated within circles. (F) Comparison between the structure of Rvb1p-Rvb2p hexamers (blue color) and Rvb1p-Rvb2p dodecamers (EMD-3080) (Ewens et al., 2016) (shown as white transparent density). The location of the Rvb2p-DII domain after the putative movement needed to accommodate to its position in Rvb1p-Rvb2p dodecamers is shown in orange color. See also Figure S2.

Cryo-EM images of glutaraldehyde-stabilized R2TP were processed first in 2D, giving side views of R2TP with density for putative TP molecules occupying a region between the DII domains (Figure 2A). A 3D structure at low resolution indicated that yeast R2TP overall does not follow 3-fold symmetry, which is restricted to the AAA+ ring, and that the putative TP component is an asymmetric entity lying within the area of the DII domains (Figure 2B). To improve the resolution, we assembled the R2TP complex in the same way as for the AUC experiments, without any cross-linking (Figures 2C and S2). We also merged untilted and tilted data (0° and 35° tilted), to increase the sampling of different views of the complex (Figure 2C).

After 3D classification, we obtained a sub-population of 24,835 particles (29% of the total dataset) that corresponded to free Rvb1p-Rvb2p hexamers that displayed a consistent C3 rotational symmetry. The R2 structure was determined at 6.5Å resolution overall (Figures S2B–S2D), but with the AAA+ core displaying higher local resolution than the DII domains, reflecting their intrinsic flexibility (Figures 2D, 2E, and S2E). An atomic model of yeast Rvb1p-Rvb2p was constructed based on the crystal structure of the highly homologous *Chaetomiun thermophilum* Rvb1-Rvb2 (Lakomek et al., 2015), and fitted into the cryo-EM map. Rvb1p and Rvb2p subunits in R2 could be identified in maps due to the extended C-terminal helix of Rvb2p compared with Rvb1p (Figures 2D and 2E). Interestingly, the different orientations of the DII domains we observe in our Rvb1p-Rvb2p complex are consistent with those described in *Chaetomiun thermophilum* (Lakomek et al., 2015) with Rvb1p-DII orientated axially and the Rvb2p-DII orientated more equatorially. This differs from the orientations observed in a lower-resolution cryo-EM reconstruction of a dodecameric assembly (Ewens et al., 2016), in which both Rvb1p and Rvb2p DII domains have a similar axial orientation (Figure 2F).

**Figure 3:**
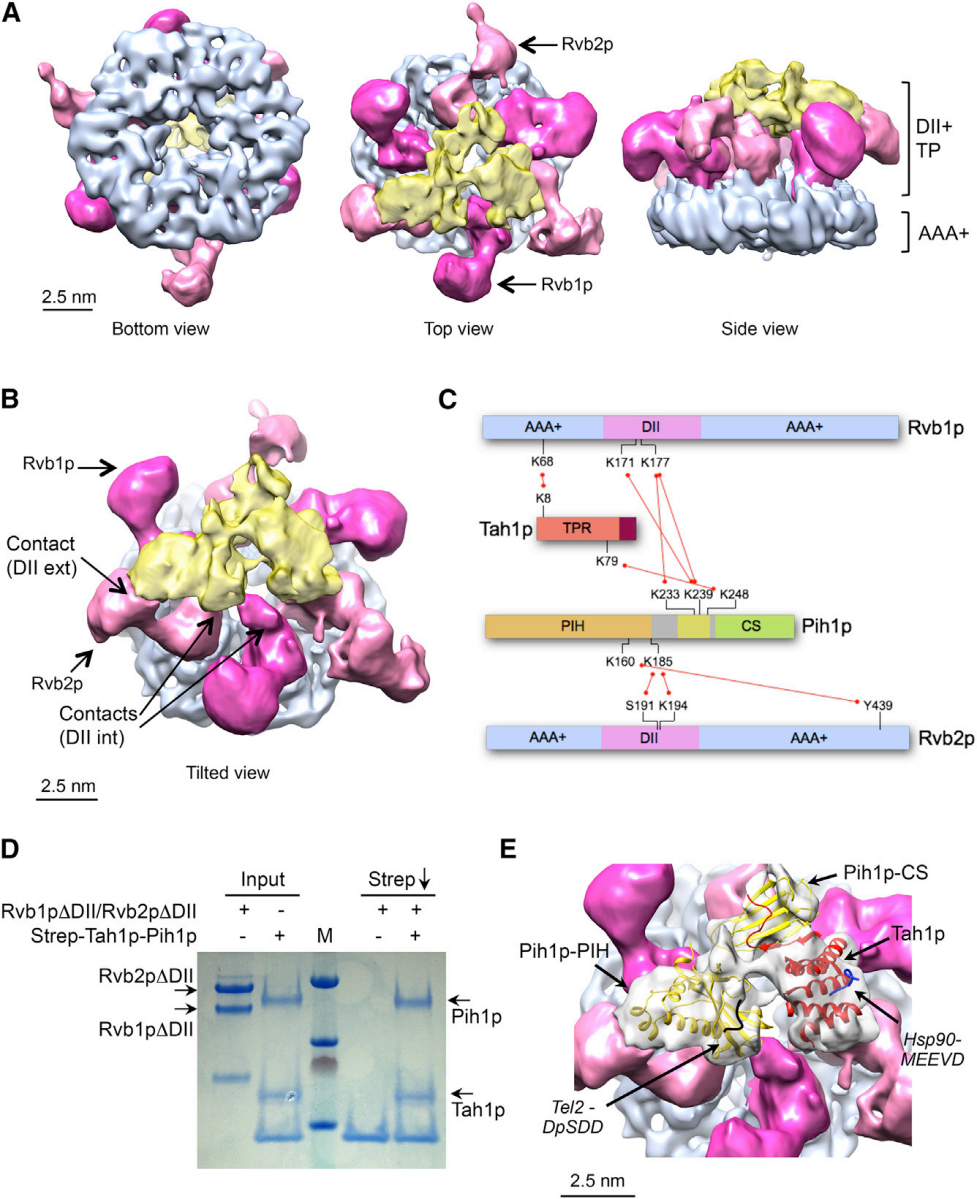
Structure of Yeast R2TP (A) Several views of the structure of yeast R2TP, with the Rvb1p-Rvb2p AAA+ ring colored in blue, DII domains in pink, and TP in yellow. Due to the differences in resolution, we used a B-factor of −400 for representation of the AAA+ ring, which is solved at higher resolution, whereas the automatic B factor calculated by Relion (−24) was used for the DII-TP. DII domains in Rvb1p and Rvb2p are colored in dark and light pink, respectively. (B) A tilted view of yeast R2TP as in (A), with contacts between TP and DII domains of R2 highlighted (int, internal and ext, external). (C) Schematic of inter-molecular cross-links identified by mass spectrometry. Intra-molecular cross-links and cross-links between Rvb1p and Rvb2p have been omitted for clarity. The cross-link patterns highlight the role of the DII domains of Rvb1p and Rvb2p (pink) and the central segment of Pih1p (yellow) in coupling the TP and R2 sub-complexes. (D) Strep-tag pull-down experiment using Strep-Tah1p-Pih1p complex and a DII-truncated version of Rvb1p and Rvb2p. M indicates Molecular Weight markers. (E) Close-up view of the fitting of Tah1p and Pih1p crystal structures in yeast R2TP. The TP density in the map is shown in transparent density where the crystal structure of the TPR domain of Tah1p (PDB: 4CGU) (red color), the CS domain (PDB: 4CGU), and PIH domain of Pih1p (PDB: 4CGW) (Pal et al., 2014) (yellow color) have been fitted. The relative orientations of the Tah1p-TPR and Pih1p-CS domains have been adjusted from the orientation observed in their co-crystal structure. The positions of the C-terminal MEEVD peptide of Hsp90 bound to Tah1p and the DpSDDE phosphopeptide of Tel2p are indicated (Pal et al., 2014). See also Figures S3 and S4.

The structure of yeast R2TP was determined at 8.37 Å resolution, without imposing symmetry. 60,293 particles were classified as R2TP (approximately 71% of the particles). As initial 3D classification revealed variability in the region comprising the TP bound to the DII domains, comparable with that seen for the DII domains in the isolated R2 structure, particles were re-classified based on the conformation of the DII region plus the TP. A subset of 38,962 particles converged into a structure consistent with a single conformation of R2TP at this resolution (Figures 3A and S3A–S3C). The AAA+ core was better defined than the DII domains-TP assembly (Figure S3D), consistent with the intrinsic flexibility of these regions.

R2TP forms an open cage (a hexameric ring open at both ends) where TP occupies most of the available space between the DII domains. TP interacts closely with R2 at the level of the DII domains (Figure 3B), coming close to the OB-fold domain (DII exterior) of one Rvb2p subunit, and to the DII region proximal to the AAA+ core (DII interior) in the other Rvb subunits in the hetero-hexamer (Figure 3B). Consistent with the apparently specific interaction of TP with the OB-fold in one Rvb2p subunit, the orientations of the DII domains of Rvb2p in the presence of TP are markedly asymmetric. Thus, the density associated with these degrades substantially if particles are processed with C3 rotational symmetry, while those of Rvb1p retain a similar orientation as in isolated R2 and are largely unaffected by symmetrization (Figure S3E).

To more precisely map contacts between the proteins, we subjected the R2TP complex to cross-linking mass spectrometry analysis (XL/MS; see STAR Methods). We observed cross-links between residues in the central region of Pih1p and residues in the DII domain of both Rvb1p and Rvb2p (Figures 3C and S4), consistent with the proximity of TP with these regions of R2 indicated by the EM maps.

We also tested R2TP assembly under several conditions in pull-down assays. When the DII domains in Rvb1p-Rvb2p were truncated, the interaction with TP is abolished (Figure 3D). In addition, Rvb1p-only and Rvb2p-only complexes showed very little interaction with TP in pull-down experiments (not shown), whereas Rvb1p-Rvb2p showed a tight interaction. Also, isolated DIIs from Rvb1p or Rvb2p did not interact with TP (not shown). These results indicate that Rvb1p and Rvb2p subunits are needed for R2TP assembly, and that the DII domains are necessary but not sufficient for the interaction.

### Structural Model of Yeast R2TP

Crystal structures for all but one of the core domains of yeast R2TP are available, and the R2TP cryo-EM structure could be readily interpreted by fitting these as rigid bodies into the cryo-EM map. The majority of the yeast TP structure has been determined at high resolution in two modules. One module consists of the N-terminal PIH domain of Pih1p (PDB: 4CHH) (Pal et al., 2014), while the other (PDB: 4CGU) contains the C-terminal CS domain of Pih1p bound to the C-terminal segment of Tah1p that extends beyond the globular TPR domain. The structure of the region connecting the PIH and CS domains of Pih1p is currently unknown.

To perform this fitting, the density of TP was extracted from the R2TP map by subtracting Rvb1p-Rvb2p density. TP within R2TP comprises two large and one smaller domain. The Pih1p N-terminal domain and Tah1p have dimensions that could only be accommodated within the two largest densities, leaving the smaller region of density for the CS domain of Pih1p (Figure 3E). The orientation of the fitted Tah1p TPR domain allows its C-terminal extension to bind to the CS domain of Pih1p, and exposes the peptide binding groove at the surface, allowing access by the C-terminal MEEVD motif of Hsp90. Similarly, the docked orientation of the PIH domain allows full access by a CK2-phosphorylated peptide such as that found in Tel2p and other putative R2TP clients (Horejsi et al., 2010, Horejsi et al., 2014, Pal et al., 2014).

**Figure 4:**
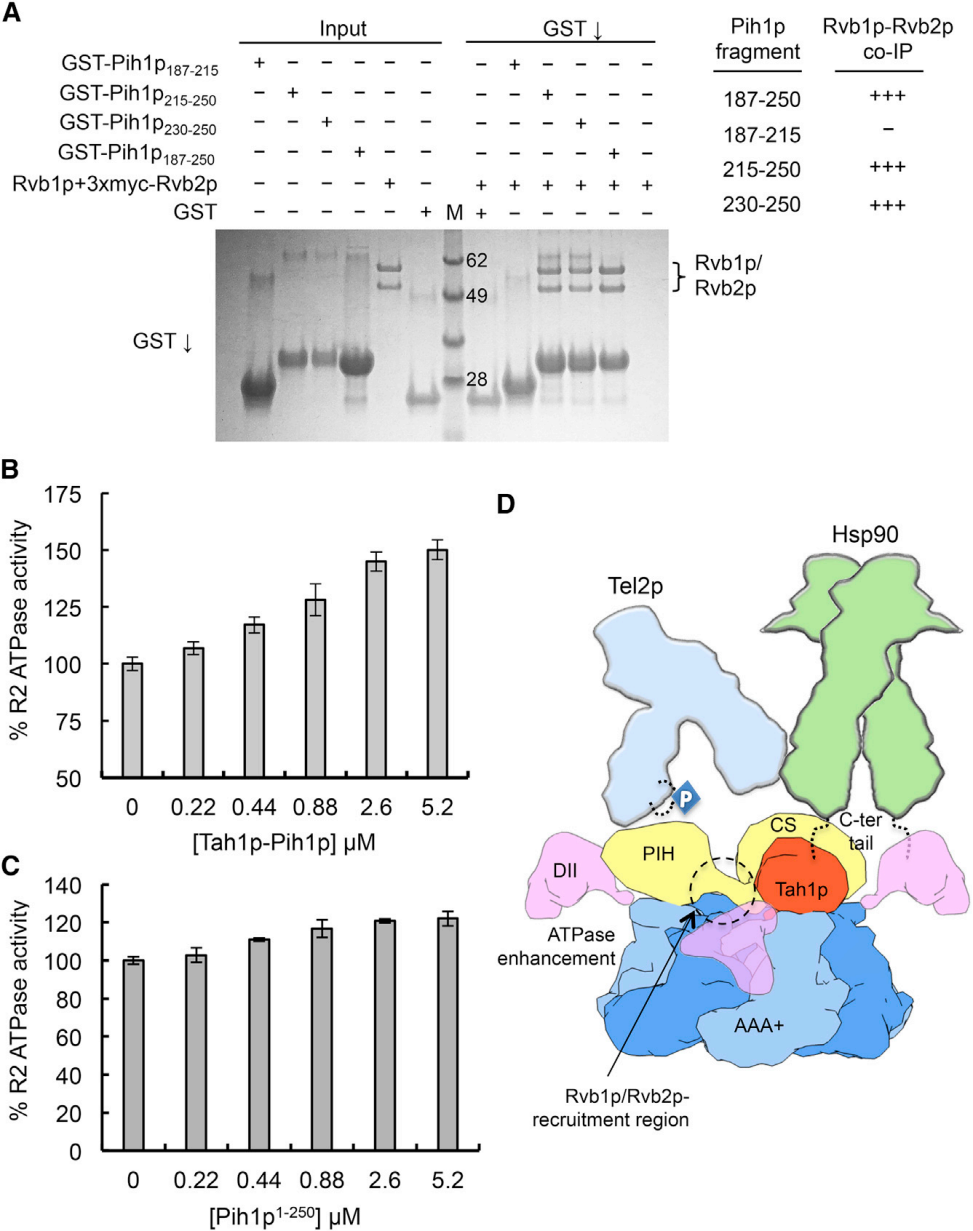
Interactions and ATPase Activity in Yeast R2TP (A) Mapping the essential interacting region of Pih1p by pull-down assay. Assembled R2 complex was incubated with various GST fusions to segments of Pih1p. Robust co-precipitation was observed with GST-Pih1p constructs that included residues 215–250 and 230–250 but were eliminated or substantially weakened when residues 215–250 were absent. M indicates Molecular Weight markers. (B) ATPase activity of Rvb1p-Rvb2p is activated by addition of the TP sub-complex. TP was added to R2 (0.5 μM) at the concentrations shown. Values are averages of three replicates and the error bars indicate 1 standard deviation (SD) around the mean value. (C) ATPase activity of Rvb1p-Rvb2p is activated by a Pih1p fragment comprising residues 1–250. Values are averages of three replicates and the error bars indicate 1 SD around the mean value. (D) Cartoon model of the Hsp90-R2TP-TTT super-complex. DII domains are disposed asymmetrically around the Rvb1p-Rvb2p basket and provide the binding sites for one Pih1p-Tah1p complex, which ensures that only a single Hsp90 dimer would be recruited. A unique region between the PIH and CS domains of Pih1p is essential for interaction with R2. The interaction of TP with the flexible DII domains enhances R2 ATPase activity. The Hsp90-binding Tah1p TPR domain and the Tel2p-binding PIH domain of Pih1p face the same side, bringing Tel2p and the Hsp90 chaperone into close proximity.

The remaining unoccupied density bridges between the Pih1p PIH and CS domains would readily accommodate the ∼80 residues that connect these domains in the primary sequence of the protein. Previous studies have suggested that residues in this region are required for interaction with R2 (Paci et al., 2012). To test this, we constructed a set of GST-fused Pih1p sub-constructs and examined their ability to co-precipitate Rvb1p and/or Rvb2p (Figure 4A). A construct containing just residues 230–250 retained the ability to bind R2. Consistent with a role in mediating Pih1p interactions with R2, residues in this region formed cross-links to Rvb1p in the XL/MS analysis (Figure 3C).

### TP Stimulates the ATPase Activity of R2

Previous studies have suggested that the conformations of the DII domains within observed dodecameric assemblies of R2 change in different adenine nucleotide-bound states (Ewens et al., 2016, Lakomek et al., 2015). As TP binding clearly affects the symmetry and orientation of the DII domains in the R2TP complex we describe here, we examined the effect of TP on the ATPase activity of R2. Using a sensitive coupled enzyme assay, we measured the basal rate for hydrolysis of ATP by yeast R2 as 0.53 mol/min/mol. Titration of increasing amounts of TP produced a roughly sigmoidal activation curve with a maximal observed ATPase stimulation of more than 50% of the basal level (Figure 4B). A Pih1p fragment containing the linker but not the CS domain (residues 1–250) was sufficient to stimulate the ATP activity although activation was weaker than when using the TP complex (Figure 4C).

## Discussion

R2TP is the most complicated of the various Hsp90 co-chaperones identified so far, being a multiprotein complex itself, and possessing components (Rvb1 and Rvb2) that like Hsp90 possess inherent ATPase activity on their own (Jeganathan et al., 2015, Nano and Houry, 2013). The role of the ATPase activity of Rvb1 and Rvb2 in the function of R2TP is very poorly understood.

Although the isolated Rvb1 and Rvb2 proteins appear to be able to form homomeric rings in vitro (Matias et al., 2006), the biologically relevant arrangement is believed to be, as observed here (Figures 2D and 2E), an alternating hetero-hexamer of Rvb1 and Rvb2 molecules. Association of these hetero-hexamers into dodecamers has been observed in several structural studies (Ewens et al., 2016, Gorynia et al., 2011, Jeganathan et al., 2015, Lakomek et al., 2015, Torreira et al., 2008), but the biological relevance of this larger form is unclear. Regardless of the behavior of the Rvb1-Rvb2 complex in isolation, in the yeast R2TP complex we have structurally characterized here, the Rvb1p and Rvb2p proteins are present unambiguously as a single alternating hetero-hexameric ring (Figure 3).

Previous crystallographic and nuclear magnetic resonance studies of the TP (TPR and PIH domain containing) components of yeast R2TP have revealed the structural basis for the interactions that couple Hsp90 to Tah1p (Back et al., 2013, Pal et al., 2014), Tah1p to Pih1p (Morgan et al., 2015, Pal et al., 2014) and Pih1p to CK2-phosphorylated motifs in proteins such as Tel2p and Mre11 (Horejsi et al., 2014, Pal et al., 2014). However, the interaction of the TP sub-complex with Rvb1-Rvb2 has not been described previously. Here, we show unequivocally, both by analytical ultracentrifugation analysis and by cryo-EM reconstruction, that the yeast R2TP complex consists of a single TP bound to a single R2 hetero-hexameric ring (Figures 1 and 3).

From our cryo-EM structure, the TP binding site on R2 is provided by the DII domains, which form an open “basket” in which the sub-complex sits. The DII domains are asymmetrically disposed around the basket with the exterior OB-fold segment of one Rvb2p-DII domain interacting with the PIH domain of Pih1p, while the rest of the TP sub-complex lies closer to the interior segments of the DII domains adjacent to the AAA+ core of the ring; this structural interpretation is fully supported by the XL/MS data (Figure 3). The interaction of TP with the conformationally flexible DII domains is most likely responsible for our observation that TP binding enhances the inherent ATPase activity of R2, whose conformation appears to be coupled to the nucleotide loading of the hetero-hexamer ring (Ewens et al., 2016, Lakomek et al., 2015).

The PIH domain of Pih1p in the R2TP complex provides a binding site for proteins containing an acidic CK2-phosphorylation motif initially identified in human TEL2 (and conserved in yeast Tel2p) (Horejsi et al., 2010) and subsequently identified in a number of other proteins (Horejsi et al., 2014, Pal et al., 2014). Tel2p in turn is believed to associate with Tti1p and Tti2p to generate the TTT complex, which interacts with PI3-kinase-like kinases such as Tor1p (Kim et al., 2013). Our cryo-EM structure defines the orientations of the Hsp90-binding Tah1p TPR domain and the Tel2p-binding PIH domain of Pih1 on the same face of the R2 ring, and thus provides a mechanism whereby R2TP fulfills its role as a co-chaperone by bringing TTT-Tor1p and the Hsp90 chaperone into close proximity (Figure 4D). The presence of a single TP sub-complex in R2TP ensures that only a single Hsp90 dimer and client protein (such as Tor1p) would be recruited.

Based on the known stoichiometry of the component proteins, the putative Hsp90-R2TP-TTT chaperone super-complex contains eight ATP-binding sites, functionally segregated into two internally cooperative modules of 2 and 6. Previous studies have suggested that binding of Tah1p-Pih1p downregulates Hsp90 inherent ATPase activity, but this effect is small, and its biological relevance is unknown (Eckert et al., 2010). We show here a larger stimulatory effect of Tah1p-Pih1p on the inherent ATPase of the Rvb1p-Rvb2p complex (Figure 4B). ATPase activity is well known to be essential to the function of Hsp90 as a molecular chaperone (reviewed in Pearl, 2016), and the ATPase activity of the Rvb1p and Rvb2p proteins has been shown to be essential to their function in sustaining yeast viability. How these two ATPase modules function together in the stabilization and activation of the R2TP-mediated clientele of the Hsp90 chaperone system remains to be determined.

## STAR★Methods

### Key Resources Table

**Table:** 

REAGENT or RESOURCE	SOURCE	IDENTIFIER
**Bacterial and Virus Strains**		

Rosetta (DE3) pLysS	MERCK (Novagen)	Cat#70956-3
**Chemicals, Peptides, and Recombinant Proteins**		

BS3-H12/D12	Creative Molecules	Cat#001SS
Glutaraldehyde Solution	Sigma Aldrich	Cat#G5882-10X1ML
Phosphoenol-Pyruvate	Sigma Aldrich	Cat#10108294001
L-Lactic Dehydrogenase from bovine heart	Sigma Aldrich	Cat#L1006
Pyruvate Kinase	Sigma Aldrich	Cat#10109045001

**Deposited Data**		

Experimental map	This paper	EMD-3677
Experimental map	This paper	EMD-3678

**Recombinant DNA**		

pRSETA	Invitrogen	Cat#V35120
pET28b	MERCK (Novagen)	Cat#69864
p3E plasmid	This paper. University of Sussex	N/A
Tah1p	This paper. University of Sussex	N/A
Pih1p	This paper. University of Sussex	N/A
Rvb1p	Genscript	N/A
Rvb2p	Genscript	N/A

**Software and Algorithms**		

Scipion	(Abrishami et al., 2015, de la Rosa-Trevin et al., 2016)	http://scipion.cnb.csic.es/m/home/
CTFFIND4	(Rohou and Grigorieff, 2015)	http://grigoriefflab.janelia.org/ctffind4
RELION	(Scheres, 2012)	http://www2.mrc-lmb.cam.ac.uk/relion/index.php/Main_Page
MotionCorr	(Li et al., 2013)	http://cryoem.ucsf.edu/software/software.html
Gctf	(Zhang, 2016)	http://www.mrc-lmb.cam.ac.uk/kzhang/
ResMap	(Kucukelbir et al., 2014)	http://resmap.sourceforge.net/
Chimera	(Pettersen et al., 2004)	https://www.cgl.ucsf.edu/chimera/
SWISS-MODEL server	(Biasini et al., 2014)	(http://beta.swissmodel.expasy.org/)
MODFIT	(Lopez-Blanco and Chacon, 2013)	http://chaconlab.org/hybrid4em/imodfit
EMAN2	(Tang et al., 2007)	http://blake.bcm.tmc.edu/emanwiki/EMAN2
XMIPP	(Scheres et al., 2008)	http://xmipp.cnb.csic.es/twiki/bin/view/Xmipp/WebHome
SEDNTERP	(Laue et al., 1992)	http://www.jphilo.mailway.com/download.htm#SEDNTERP
HYDROMIC	(de la Torre et al., 2001)	http://leonardo.inf.um.es/macromol/programs/programs.htm
xQuest	(Leitner et al., 2014)	http://prottools.ethz.ch/orinner/public/htdocs/xquest/
xiNET	(Combe et al., 2015)	http://crosslinkviewer.org/

**Other**		

Talon	Clontech	Cat#635504
GST beads	GE Healthcare	Cat#17527902
Vivaspin MW Cut-Off 10K	Sartorius	Cat#VS0602
Superdex S200 10/300 GL	GE Healthcare	Cat#17517501
Streptavidin Magnetic Beads	NEB Inc.	Cat#S1420S
StrepTactin Sepharose High Performance Resin	GE Healthcare	Cat#28-9355-99
HiPrep Sephacryl S-200 HR	GE Healthcare	Cat#17119501
R 2/2 300 mesh Cu holey carbon grids	Quantifoil	N/A
Superdex Peptide 3.2/300	GE Healthcare	Cat#29036231

### Contact for Reagent and Resource Sharing

Further information and requests for resources and reagents should be directed to and will be fulfilled by the Lead Contact, Oscar Llorca (ollorca@cnio.es).

### Experimental Model and Subject Details

#### Bacterial Strains Used for Protein Expression

The Tah1p gene was cloned into NdeI and BamHI sites of a modified pET28b plasmid that resulted in 2xStrep-tagged Tah1p protein. The yeast PIH1P gene was cloned into a p3E plasmid (modified pGEX6p in our lab) using NdeI and HindIII sites. The Rvb1p gene was cloned using NdeI and BamHI sites and Rvb2p using NheI and HindIII sites into pET28b and modified pRSETA plasmid (containing 3X Myc-tags) respectively. The PIH1_187-250_ (Rvb binding domain) gene was cloned into p3E plasmid using NdeI and BamHI sites. The Tah1p-Pih1p, the Rvb1p-Rvb2p protein complexes were co-expressed in Rosetta (DE3) *pLysS* cells.

### Method Details

#### Protein Purification

The cells were lysed using a sonicator in 20 mM HEPES pH 7.5, 500 mM NaCl. The cell lysate was centrifuged at 18,500 g for an hour. The clear supernatant was added to equilibrated Talon or GST beads for affinity chromatography. The proteins were eluted using imidazole or glutathione from the Talon or GST beads respectively. The protein complexes were further purified by gel filtration using S200 26/60 column.

#### Pull Down Assay

20 μM GST-Pih1_187-250_ was mixed with 30 μl GST beads, which were equilibrated in 50 mM HEPES pH 7.5, 140 mM NaCl. 60 μM of the Rvb1p-Rvb2p complex was added to the above mixture. This complex was incubated for 45 min at 4°C rotating at 20 rpm/min. The beads were washed three times with 500 μl of 20 mM HEPES pH 7.5, 140 mM NaCl buffer and the bound fraction was eluted using 50 mM glutathione.

For the full-length R2TP complex pull-down assay, all complexes (Tah1p-Pih1p and Rvb1p-Rvb2p) were diluted in 20 mM HEPES pH 7.5, 140 mM NaCl. 60 μM of the Rvb1p-Rvb2p complex was added to 20 μM 2xStrep-Tah1p-Pih1p complex. The final complex was mixed with 30 μl anti-strep magnetic beads (NEB Inc.) and incubated for 45 min rotating at 20 rpm at 4°C. The beads were washed three times with 500 μl of 20 mM HEPES pH 7.5, 140 mM NaCl buffer. The bound fraction was eluted with 2.5 mM desthiobiotin in 20 mM HEPES, pH 7.5, 140 mM NaCl.

#### yRvb1DL-yRvb2DL Expression, Purification and Its Interactions with Tah1p-Pih1p

2X-Flag-tagged-Rvb2p without domain II (Rvb2pΔII) was cloned into NheI and BamHI of pRSETA and Rvb1p without domain II (Rvb1pΔII) into pET28b using NdeI and BamHI sites. The proteins were expressed in Rosetta DE3 pLysS cells. The cells were lysed in 50 mM HEPES pH 7.5, 140 mM NaCl and the proteins were purified using Talon affinity chromatography and SEC using S200 10/300 column (GE Healthcare). The proteins were concentrated using 10K molecular weight cut-off Vivaspin. Both Rvb1pΔII/Rvb2pΔII and 2XStrep-Tah1p-Pih1p were dialysed in 50 mM HEPES pH 7.5, 140 mM NaCl. 50 μl anti-strep magnetic beads (NEB Inc.), which were equilibrated in 50 mM HEPES pH 7.5, 140 mM NaCl, mixed with 50 μM of each of Rvb1pΔII/Rvb2pΔII and 2XStrep-Tah1p-Pih1p protein complex. The mixture was incubated by rotating at 20 rpm/min at 4°C for 45 min. The beads were washed three times with 500 μl of 20 mM HEPES pH 7.5, 140 mM NaCl, 0.01 % Tween20 buffer and the bound fractions was eluted using 2.5 mM desthiobiotin in 20 mM HEPES, pH 7.5, 140 mM NaCl. The results of the pull-down assay were analysed using 4-12 % SDS-PAGE.

#### *In Vitro* Pull-Down Experiments for Negative Stain EM

Purified Tah1p-Pih1p and Rvb1p-Rvb2p complex were incubated together in a 10:1 molar ratio, respectively, for 90 min at room temperature (RT) in binding buffer (50 mM HEPES pH 7.8, 200 mM NaCl, 0.5 mM TCEP, 0.5 mM TX-100) in a final volume of 60 μl. The mixture was then incubated with StrepTactin Sepharose High Performance Resin (GE Healthcare) for 30 min at RT, and the unbound material was removed by centrifugation. After three washing steps the sample was eluted in binding buffer plus 2.5 mM desthiobiotin and cross-linked in 0.05 % glutaraldehyde for 4 min at 25°C. The reaction was terminated by the addition of 1 M Tris-HCl, pH 7.8.

5 μl of the cross-linked R2TP at a concentration of 0.03 mg/ml were adsorbed on carbon-coated grids, stained using 2 % uranyl acetate and observed in the microscope operated at 100 kV (JEOL-1230). Images were taken in low dose conditions using a TVIPS F416 CMOS at a nominal magnification of 40,000x corresponding to 2.84 Å/pixel at the specimen level. 4,536 particles were automatically picked and extracted using EMAN2 (Tang et al., 2007) and further classified and averaged using methods implemented in XMIPP (Scheres et al., 2008).

#### Sedimentation Velocity Assays

Samples at concentrations ranging from 2 to 4 μM, in 20 mM HEPES pH 7.8, 140 mM NaCl, 5 mM MgCl_2_, 0.5 mM EDTA, 0.5 mM TCEP, were loaded (320 μL) into analytical ultracentrifugation cells. The experiments were carried out at 43,000 rpm in a XL-I analytical ultracentrifuge (Beckman-Coulter Inc.) equipped with both UV-VIS absorbance and Raleigh interference detection systems, using an An-50Ti rotor, and 12 mm Epon-charcoal standard double-sector centerpieces. Sedimentation profiles were recorded at 233 nm. Differential sedimentation coefficient distributions were calculated by least-squares boundary modelling of sedimentation velocity data using the continuous distribution *c*(*s*) Lamm equation model as implemented by SEDFIT (Schuck, 2000). These *s* values were corrected to standard conditions (water, 20°C, and infinite dilution) using the program SEDNTERP (Laue et al., 1992) to obtain the corresponding standard *s* values (*s*_20, *w*_). Experimental s-values for these complexes were then correlated with the theoretical s-values obtained by using the HYDROMIC software (de la Torre et al., 2001), which predicts hydrodynamic properties of macromolecules whose three-dimensional reconstructions have been generated from electron microscopy images.

#### Cryo-EM Sample Preparation and Data Collection

Preliminary cryo-EM analysis was done on R2TP complex immediately after cross-linking. 3 μl of the stabilized complex were applied to glow-discharged holey-carbon (Quantifoil) grids on which an extra thin layer of home-made carbon had previously been deposited, and the grids were vitrified using a Vitrobot (FEI). The grids were observed in a Titan Krios (FEI) operated at 300 kV and coupled to a Falcon II camera (FEI) (https://www.ceitec.eu/z7, CEITEC, Brno). Images were recorded at a nominal magnification of 59,000x that corresponds to 1.38 Å/pixel at the specimen level and with a 2-3.5 μm range of defocus. Frames were recorded during 1.5-s exposures that correspond to an accumulated total dose of 42 e^-^/pixel.

For R2TP without cross-linking, Tah1p-Pih1p and Rvb1p-Rvb2p were incubated together in a 5:1 molar ratio respectively while dialysing against 500 ml of 20 mM HEPES pH 7.8, 140 mM NaCl, 5 mM MgCl_2_, 0.5 mM EDTA, 0.5 mM TCEP buffer for 1 h at 25°C in order to lower the salt content. The mixture was recovered and ADP was added to a final concentration of 1 mM. 3 μl of the complex at a concentration of 0.45 mg/ml were then applied to holey-carbon (Quantifoil) grids and vitrified using a Vitrobot (FEI). Grids were observed in a Titan Krios (FEI) operated at 300 kV and equipped with a Quantum K2 Summit in counting mode (Gatan) and a Gatan Image Filter (GIF) with a slit width of ∼20eV (eBIC-Diamond, http://www.diamond.ac.uk, Oxford). Data was recorded on counting mode at a nominal magnification of 39,000x that corresponds to 1.34 Å/pixel at the specimen level. Two types of datasets with different tilts were collected (0° and 35°). For the 0° dataset the range of defocus was 1.5-3 μm, and 12-s exposures with a total accumulated dose of 48 electrons per Å^2^ were taken in 30 frames. For the 35° dataset the range of defocus was 2-3 μm and the same total exposure was fractionated in 40 frames.

#### Cryo-EM Data Processing

For the dataset collected at CEITEC a total of 3,595 movies were collected. Frame alignment in each movie stack was performed using the combined cross-correlation and optical flow approach available in Scipion (Abrishami et al., 2015, de la Rosa-Trevin et al., 2016). The CTF parameters from the summed micrographs containing all the frames were determined using CTFFIND4 (Rohou and Grigorieff, 2015). 287,620 particles were initially selected and subjected to 2D and 3D classification using RELION-1.3 (Scheres, 2012). The starting model for 3D classification was low-pass filtered to 40 Å, and was built combining characteristic top and side views of the negatively stained Rvb1p-Rvb2p complex. The 3D classification led to a homogeneous subset of 26,781 that was further refined to a consensus map at 11.5 Å resolution without applying symmetry (gold-standard FSC_0.143_).

For the dataset collected at eBIC-Diamond a total of 1,745 movies were collected. MotionCorr was used to correct the beam-induced sample motions in each movie stack (Li et al., 2013) and the summed micrographs containing all the frames were used for further processing. CTF parameters were determined and locally refined using Gctf (Zhang, 2016). 346,693 particles were initially selected using the automatic particle picking and processed using routines available in RELION-1.3 (Scheres, 2012). These particles were subjected to manual inspection, 2D and 3D classification imposing no symmetry, what produced a ‘cleaned-up’ set of 85,128 particles. The starting model for 3D classification was low-pass filtered to 40 Å and corresponded to the consensus map previously obtained using data collected at CEITEC. The ‘cleaned-up’ set was further classified based on the occupancy of the Rvb1p-Rvb2p ring, which led to two different subsets of particles corresponding to Rvb1p-Rvb2p and R2TP complex. The Rvb1p-Rvb2p subset was refined imposing C3 symmetry giving a map at 6.5-Å resolution (gold-standard FSC_0.143_) from 24,835 particles. The subset of particles corresponding to the R2TP was further classified based on a soft 3D mask corresponding to the extra density of the TP. A homogeneous set of particles was then selected and further refined using no symmetry in order to obtain the final structure of the R2TP. This structure is made of 38,962 particles and has a resolution of 8.37-Å (gold-standard FSC_0.143_). The final refinements of the Rvb1p-Rvb2p and R2TP maps were performed with particles from summed micrographs with a total accumulated dose of 30 electrons per Å^2^. These micrographs come from summing frames 2-19 and 2-26 from 0° and 35° movie stacks respectively. The sharpening of the refined structures was achieved by using automatic B-factor estimation within RELION 1.3 (Scheres, 2012). For R2TP, different B-factors were used for representation in Figures, as indicated in the legend of the Figure, due to the different resolutions in the AAA+ and TP sections of the map. Local resolution was estimated using ResMap (Kucukelbir et al., 2014). Structures were visualised using UCSF Chimera (Pettersen et al., 2004).

#### Model Generation, Fitting and Visualization

The atomic model for the hexameric Rvb1p-Rvb2p complex from *Saccharomyces cerevisiae* was initially built using the beta-oligo-version of the SWISS-MODEL server ((http://beta.swissmodel.expasy.org/)(Biasini et al., 2014). A new functionality of this version allows modelling of the oligomeric form of the target sequence based on the quaternary structure of the template. Template structures for model building correspond to PDB files 4WW4 (Rvb1-Rvb2 from *Chaetomium thermophilum*) and 2C9O (human RUVBL1). The atomic model was initially fitted using the rigid body approach available in UCSF Chimera (Pettersen et al., 2004), and further refined using the flexible fitting software iMODFIT (Lopez-Blanco and Chacon, 2013). EM maps and atomic models were visualized using UCSF Chimera (Pettersen et al., 2004). Fitting of crystal structures of the CS domain of Pih1p bound to Tah1p (PDB 4CGU) and the N-terminal PIH domain of Pih1p (PDB 4CHH) (Pal et al., 2014) were fitted into R2TP EM map manually using UCSF Chimera (Pettersen et al., 2004).

#### ATPase Assays

ATPase assays were conducted in triplicate at 37^o^C. Each 1 ml assay contained 100 μL of 10 x buffer (4.8 ml 1M Hepes pH 7.5, 1 mL 5 M NaCl and 0.2 ml 1 M MgCl_2_), 200 μL 10 mM ATP, 100 μL 10 mM phosphoenol pyruvate, 12 μL 50 mM NADH, 4 μL lactate dehydrogenase (Sigma), 20 μL pyruvate kinase (Roche), 0.5 μM R2 complex (concentration based on a dimer unit of Rvb1p-Rvb2p). TP complex or a fragment of yeast Pih^1-250^, representing amino acids 1-250 were added to the assay at 0.22, 0.44, 0.88, 2.6 and 5.2 μM (based on a heterodimer complex of TP or yeast Pih^1-250^) and distilled H_2_O to 1 ml.

#### Cross-Linking Mass Spectrometry

A purified solution of yeast R2TP complex at a concentration of 1 mg/ml was cross-linked using a homobifunctional, isotopically-coded N-HydroxySuccinimide (NHS) ester BS3 (H12/D12) purchased from Creative Molecules (Canada) at a concentration of 0.192 mg/ml and 0.096 mg/ml.

Cross-linked samples were lyophilised and then resuspended in 50 mM NH_4_HCO_3_ to a final protein concentration of 1 mg/ml, prior to reduction with 10 mM DTT and alkylation with 55 mM iodoacetamide. Following alkylation, proteins were digested with Trypsin (Promega, UK) at an enzyme-to-substrate ratio of 1:20, overnight at 37°C. The resulting peptides were acidified with formic acid and fractionated by peptide size exclusion chromatography, using a Superdex Peptide 3.2/300 (GE Healthcare) with a 30 % v/v Acetonitrile 0.1% v/v TFA mobile phase at a flow rate of 50 ul/min. Fractions were collected every 2 min from the elution volume 1.0 ml to 1.7 ml. Prior to LC-MS analysis fractions were lyophilised and resuspended in 2 % v/v Acetonitrile and 2 % v/v formic acid.

The digests were analysed by nano-scale capillary LC-MS/MS using an Ultimate U3000 HPLC (ThermoScientific Dionex, San Jose, USA) to deliver a flow of approximately 300 nL/min. A C18 Acclaim PepMap100 5 μm, 100 μm x 20 mm nanoViper (ThermoScientific Dionex, San Jose, USA), trapped the peptides prior to separation on a C18 Acclaim PepMap100 3 μm, 75 μm x 250 mm nanoViper (ThermoScientific Dionex, San Jose, USA). Peptides were eluted with a gradient of acetonitrile. The analytical column outlet was directly interfaced via a nano-flow electrospray ionisation source, with a hybrid dual pressure linear ion trap mass spectrometer (Orbitrap Velos, ThermoScientific, San Jose, USA). Data dependent analysis was carried out, using a resolution of 30,000 for the full MS spectrum, followed by ten MS/MS spectra in the linear ion trap. MS spectra were collected over a m/z range of 300–2000. MS/MS scans were collected using threshold energy of 35 for collision-induced dissociation.

Thermo Xcalibur .raw files were converted into the open mzXML format through MSConvert (Proteowizard) with a 32-bit precision. mzXML files were directly used as input for xQuest (Leitner et al., 2014)searches on a local xQuest installation. The selection of cross-link precursors MS/MS data was based on the following criteria: 12.07532 Da mass difference among the heavy and the light BS3; precursor charge ranging from 3+ to 8+; maximum retention time difference 2.5 min. Searches were performed against an ad-hoc database containing the sequences of all yeast R2TP subunits together with their reverse used as decoy. The following parameters were set for xQuest searches: maximum number of missed cleavages (excluding the cross-linking site) 2; peptide length 4–50 amino acids; fixed modifications carbamidomethyl-Cys (mass shift 57.02146 Da); mass shift of the light cross-linker 138.06808 Da; mass shift of mono-links 156.0786 and 155.0964 Da; amino acids required K, S and Y; MS1 tolerance 10 ppm, MS2 tolerance 0.2 Da for common ions and 0.3 for cross-link ions; search in enumeration mode (exhaustive search). Search results were filtered according to a MS1 mass tolerance window –3 to 7 ppm and finally each MS/MS spectra was manually inspected and validated. Cross-link network maps were created with xiNET (Combe et al., 2015).

### Data and Software Availability

The structures determined by cryo-EM have been deposited in the EMDB with accession codes EMD-3677 and EMD-3678, for yeast Rvb1p-Rvb2p and yeast R2TP respectively.

## Author Contributions

A.R.-C., M.P., C.P., L.H.P., and O.L. designed the research; M.P. expressed and purified all the individual components of the yeast R2TP and performed domain-mapping pull-down assays; C.P. performed the ATPase assays. A.R.-C. performed all EM analysis and solved the structures; J.R.L. performed the analytical ultracentrifugation experiments and analyzed the data. M.S. and G.D. performed the cross-linking mass spectrometry and analyzed the data. D.G.C. helped in the preparation of cryo-EM grids. H.M.H. helped in image processing. O.L., C.P., and L.H.P. are all equal corresponding authors; they all supervised all the work and wrote the paper, but O.L. focused more on the cryo-EM experiments, and C.P. and L.H.P. focused on biochemical and functional experiments.

## Accession Numbers

The EM map of yeast Rvb1p-Rvb2p and yeast R2TP have been deposited in the EMDB with accession codes EMD-3677 and EMD-3678.

## Supplemental Information

Document S1. Figures S1–S4

Document S2. Article plus Supplemental Information
