# Supplementary material for: The Structure of the R2TP Complex Defines a Platform for Recruiting Diverse Client Proteins to the HSP90 Molecular Chaperone System
Source: Structure. 2017 Jul 5;25(7):1145–1152.e4. doi: 10.1016/j.str.2017.05.016 (PMC5501727; doi:10.1016/j.str.2017.05.016)
Supplement: Document S1. Figures S1–S4 [file mmc1.pdf]

Structure, Volume *25*

Supplemental Information

The Structure of the R2TP Complex Defines a Platform for Recruiting Diverse Client Proteins to the HSP90 Molecular Chaperone System

Angel Rivera-Calzada, Mohinder Pal, Hugo Muñoz-Hernández, Juan R. Luque-Ortega, David Gil-Carton, Gianluca Degliesposti, J. Mark Skehel, Chrisostomos Prodromou, Laurence H. Pearl, and Oscar Llorca

Supplemental Figures and legends

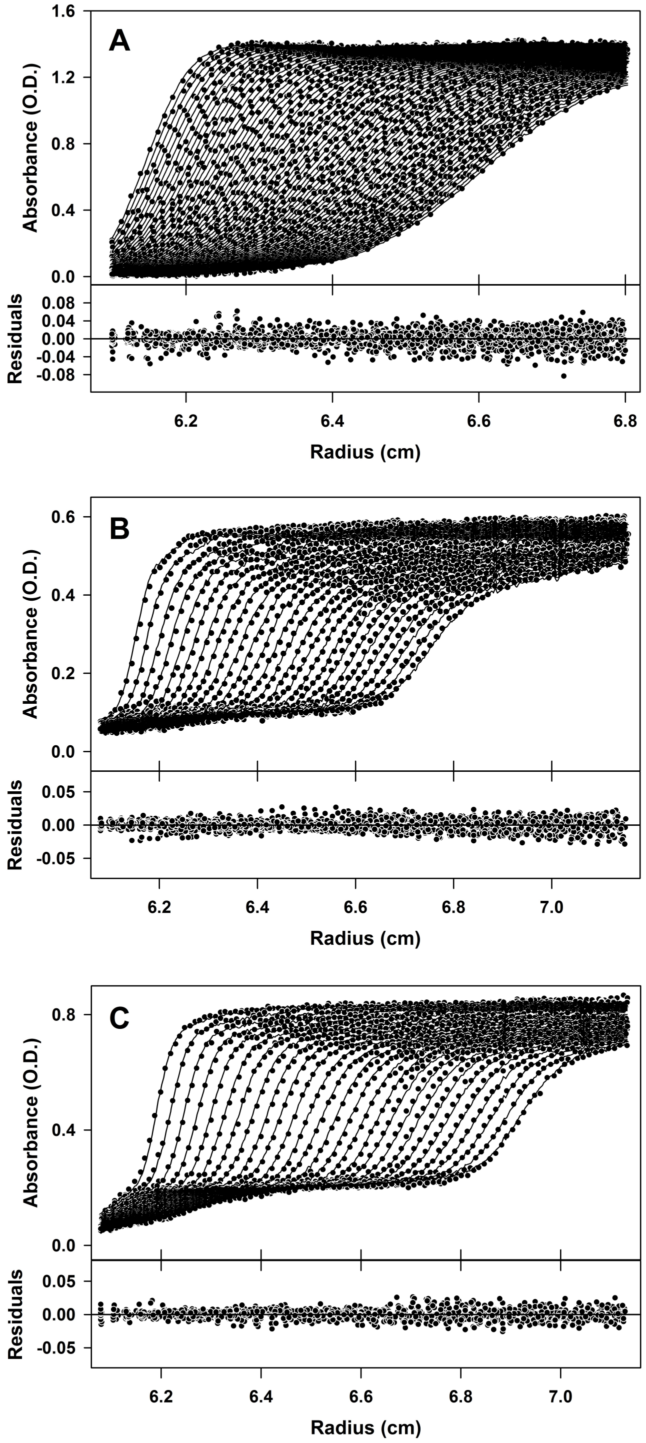

Figure S1. Sedimentation velocity boundaries. Related to Figure 1. Experimental data (solid circles) and their best-fit distribution from the c(s) analysis (solid lines) corresponding to purified Tah1p-Pih1p (**A**), Rvb1-Rvb2 (**B**) and the mixture of both complexes (**C**). Lower part of each plot represents the difference between experimental data and the best fit (residuals). Absorbance data in B and C were obtained with 3 mm path length centrepieces.

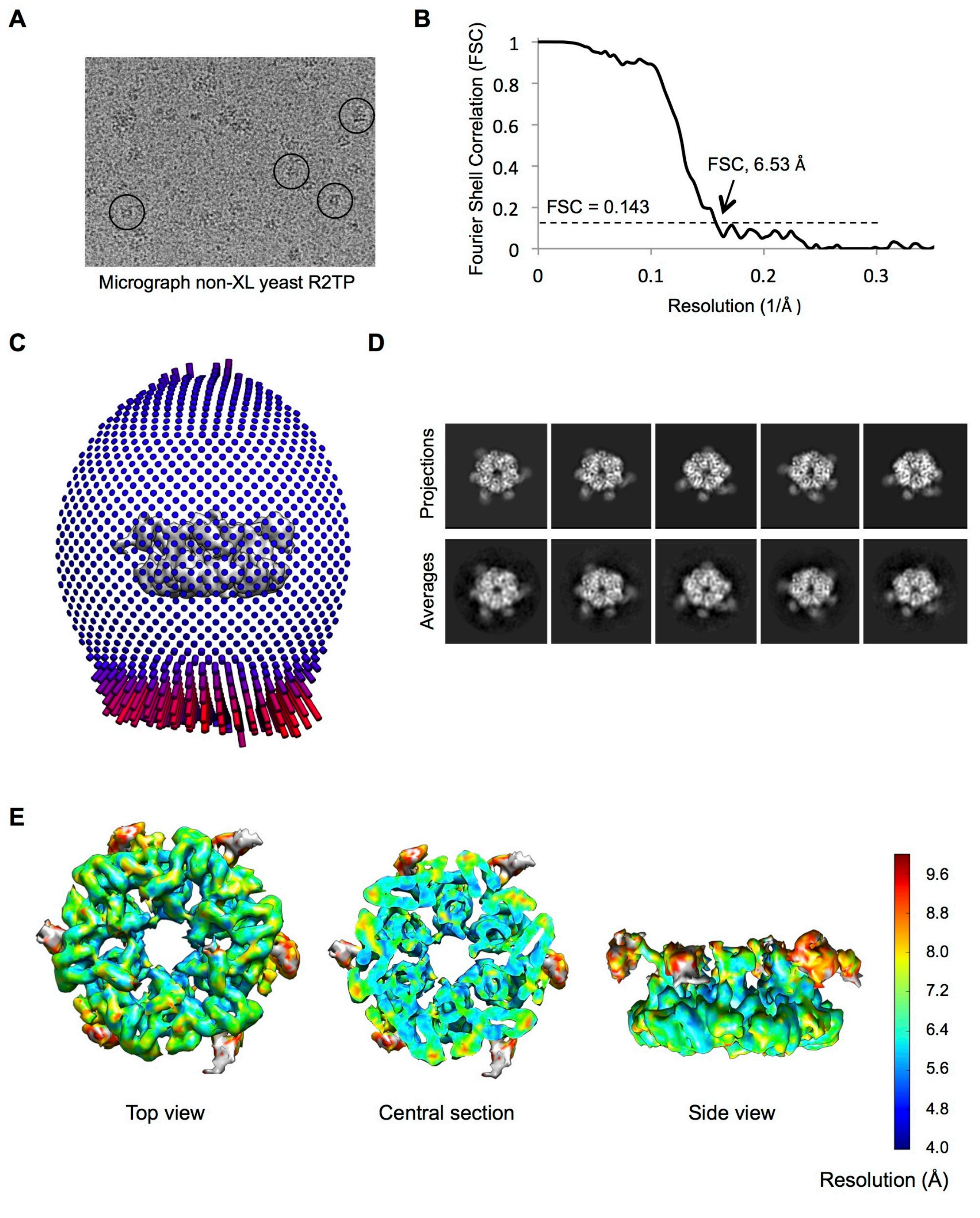

Figure S2. Cryo-EM of R2TP and 3D structure of Rvb1-Rvb2. Related to Figure 2.

A. Representative cryo-EM micrograph of yeast R2TP without cross-linking. Some background in the micrographs was due to the excess of TP in the incubation reaction. XL stands for "cross-linked".

B. Resolution estimation for the structure of Rvb1p-Rvb2p using Fourier Shell Correlation (FSC).

C. Angular distribution plot of Rvb1p-Rvb2p data set collected in vitreous ice and used in the final reconstruction. C3 symmetry was used in this reconstruction.

D. Gallery of selected reference-free 2D averages of Rvb1p-Rvb2p showing different views, compared to projections of the final structure.

E. Several views of the final structure of Rvb1p-Rvb2p coloured according to local resolutions estimated with Resmap (Kucukelbir et al., 2014) and using the scale shown in the right.

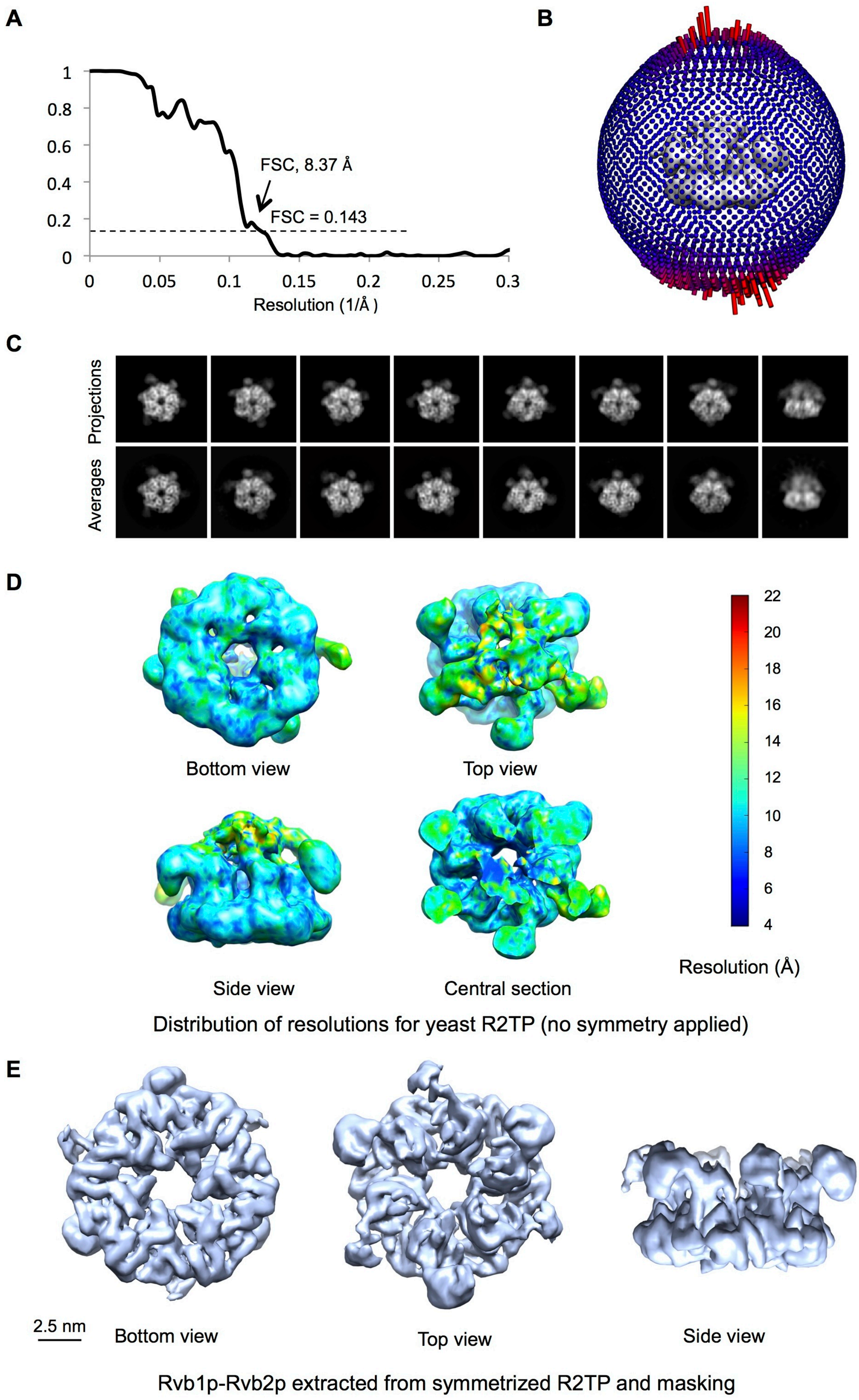

Figure S3. 3D structure of yeast R2TP. Related to Figure 3.

A. Resolution estimation for the structure of yeast R2TP using Fourier Shell Correlation (FSC).

B. Angular distribution plot of yeast R2TP data set collected in vitreous ice and used in the final reconstruction.

C. Gallery of selected reference-free 2D averages of yeast R2TP showing different views, compared to projections of the final structure.

D. Several views of the final structure of yeast R2TP coloured according to local resolutions estimated with Resmap (Kucukelbir et al., 2014) and using the scale shown in the right.

E. 3D structure of R2 hexamers within R2TP, obtained by processing the data using C3 rotational symmetry and applying a mask to remove the contribution of TP.

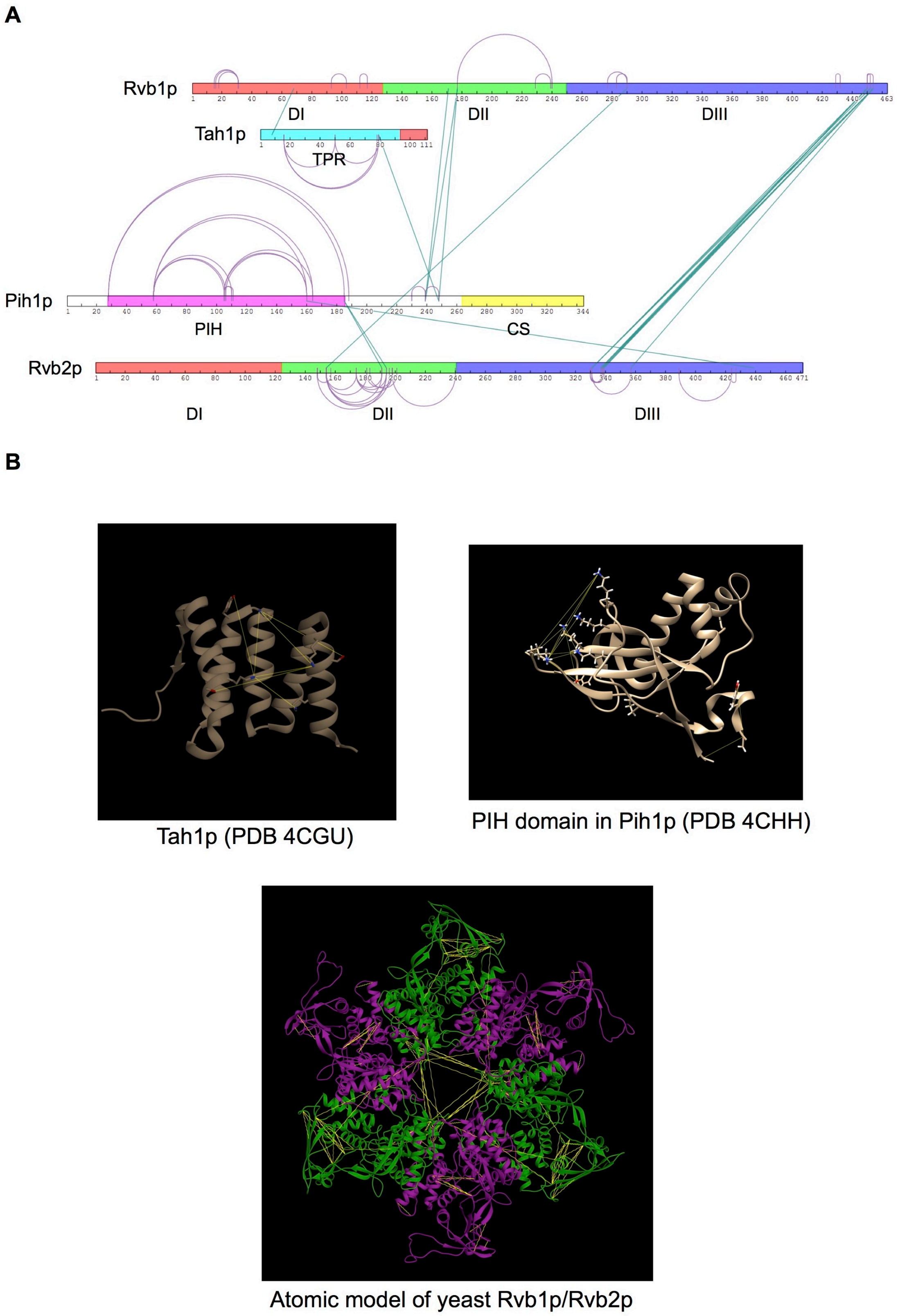

Figure S4. Mass Spectrometry Cross-link Analysis. Related to Figure 3.

A. Map of cross-links identified in the yeast R2TP complex using the cross-linker BS3 at 100 x the total protein concentration. DI, DII and DIII domains in Rvb1p and Rvb2p are coloured differently. The TPR domain in Tah1p and PIH and CS domains in Pih1p are also shown in different colour.

B. Panels showing some examples of crosslinks in the XL-MS data that are between residues that are known to be proximal for the existing structures of Tah1p-Pih1p and Rvb1p/Rvb2p. Crosslinks are represented as yellow lines, on the structures of Tah1p (PDB 4CGU) and the PIH domain in Pih1p (PDB 4CHH) (Pal et al., 2014), and also on a model of yeast Rvb1p-Rvb2p constructed based on the crystal structure of *Chaetomiun thermophilum* Rvb1-Rvb2 (Lakomek et al., 2015).
